# Pharmacologic pain management strategies for reducing postoperative pain in total knee arthroplasty: a systematic review from molecular mechanisms to clinical efficiency

**DOI:** 10.1007/s00402-025-06049-7

**Published:** 2025-09-01

**Authors:** Anca Maria Pop, Michael T. Hirschmann

**Affiliations:** 1 Department of Clinical Research, Research Group Michael T. Hirschmann, Regenerative Medicine & Biomechanics, University of Basel, Basel, Switzerland; 2 University Department of Orthopaedic Surgery and Traumatology, Kantonsspital Baselland Standort Bruderholz, Bruderholz, Switzerland

**Keywords:** Total knee arthroplasty, Postoperative pain, Chronic pain, Multimodal analgesia

## Abstract

**Introduction:**

The aim of this systematic review was to evaluate the efficiency of different analgetic regimes used in clinical practice in reducing postoperative pain and cumulative opioid consumption following total knee arthroplasty (TKA).

**Materials and methods:**

A systematic search was conducted on PubMed, Embase and Scopus according to PRISMA guidelines in order to identify appropriate studies published between 2010 and 2025, which investigated different oral or intravenous analgesic strategies (duloxetine, acetaminophen, corticosteroids, opioids, nonsteroidal anti-inflammatory drugs (NSAIDs) and gabapentinoids) in populations of patients receiving TKA by having as primary outcome the quantification of postoperative pain scores or opioid consumption.

**Results:**

Out of the 1069 identified articles, 63 met the inclusion criteria. Duloxetine improved pain scores following TKA and reduced opioid consumption, however without reaching clinical relevance. Acetaminophen, despite moderate evidence for its efficiency, remains one of the most commonly used analgesics following TKA. Gabapentinoids are useful in reducing chronic neuropathic pain, but lack efficiency in the acute clinical setting. Opioids, although highly prescribed, fail to demonstrate a clinical benefit. Intravenous corticosteroids can also provide significant pain relief due to extensive anti-inflammatory properties, while NSAIDs remain one of the mainstays of treatment due to the relevant opioid-sparing effect and acceptable safety profile.

**Conclusions:**

The appropriate management of postoperative pain following TKA relies on a multimodal approach, which emphasizes the predominant use of non-opioid analgesics. NSAIDs and acetaminophen remain validated treatments, while the applicability of other alternative agents requires further exploration in large studies.

**Supplementary Information:**

The online version contains supplementary material available at 10.1007/s00402-025-06049-7.

## Introduction

Knee osteoarthritis is a major cause of disability worldwide [1], with an expected dramatic increase of 75% in incidence by 2050 compared to 2020 [2]. Despite several available non-surgical treatment options, total knee arthroplasty (TKA) remains the mainstay of treatment in end-stage knee osteoarthritis by providing long-term pain relief and improved quality of life [3–6]. A considerable number of patients experience persistent pain after TKA, which is in contrast to total hip arthroplasty [7–10]. A recent study showed that the most common reason for early presentation to the emergency department following TKA remains uncontrolled pain with a prevalence of 15% [11], a fact that becomes even more relevant in the time of fast-track or outpatient TKA.

Various factors have been associated with the development of postoperative pain following TKA. For example, inflammatory markers such as histamine, tumor necrosis factor-α or interleukin-1β released as response to surgical trauma cause a peripheral sensitization leading to further expression of interleukin-1β in the posterior horn of the spinal cord [12]. This markedly increased inflammatory reaction leading to a later central sensitization facilitates the transition to the occurrence of chronic pain [13]. The lesion of the infrapatellar branch of the saphenous nerve is also a recognized cause of neuropathic pain following TKA [10]. Moreover, intraoperative factors such as the type of anesthesia with spinal anesthesia providing more pain comfort, type of incision, use of tourniquet, preemptive analgesia might influence the postoperative pain level [14]. Among this multitude of factors, the severity of pain in the acute postoperative phase was found to be clinically correlated with the occurrence of chronic postoperative pain, which is defined as the persistence of pain for more than 3 months [15–17].

**Fig. 1:**
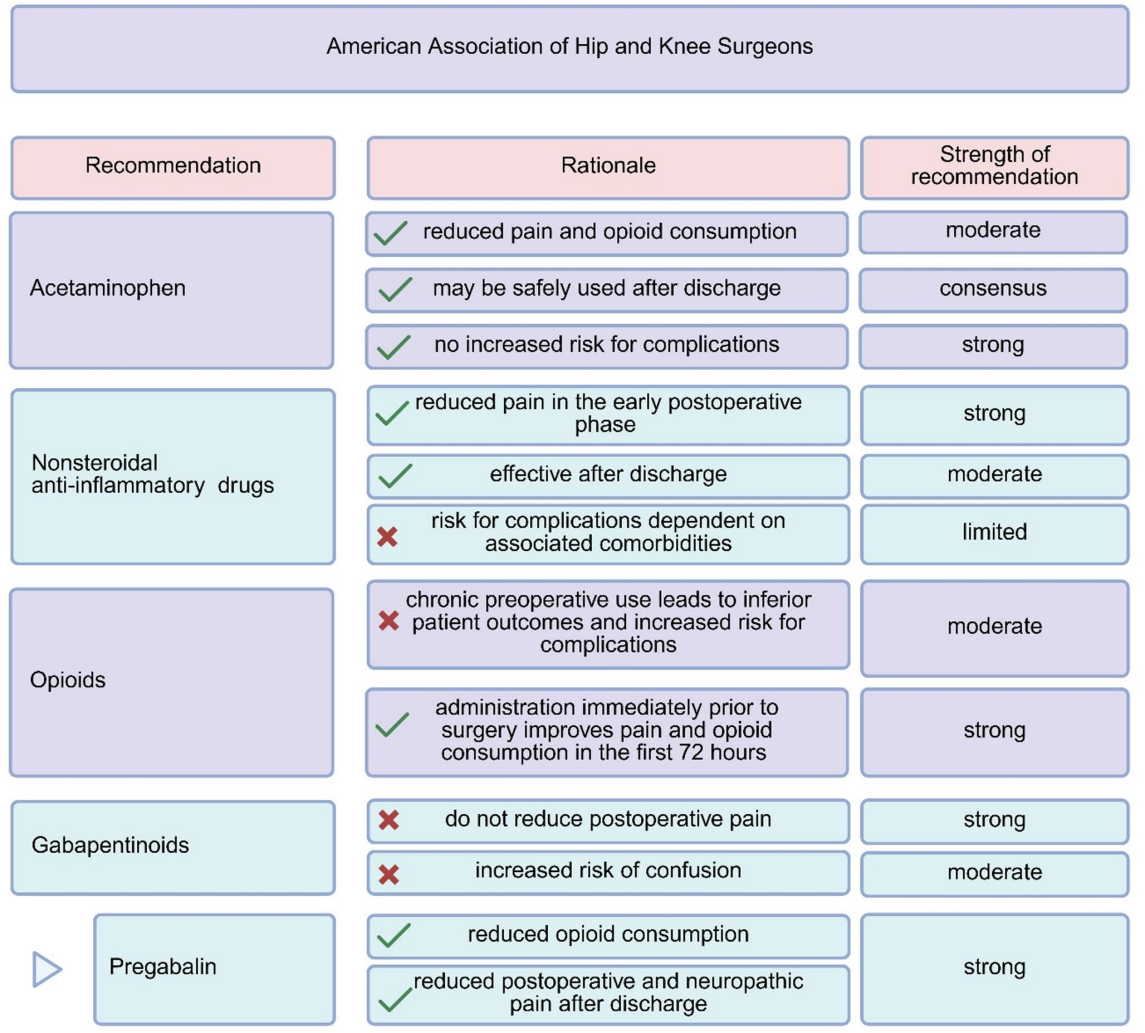
Summary of the pain management options recommended by the American Association of Hip and Knee Surgeons following total joint arthroplasty (Adapted after Fillingham et al. [18, 19] and Hannon et al. [20, 21]) Created by the Authors in BioRender (https://BioRender.com/y053ptc)

**Fig. 2:**
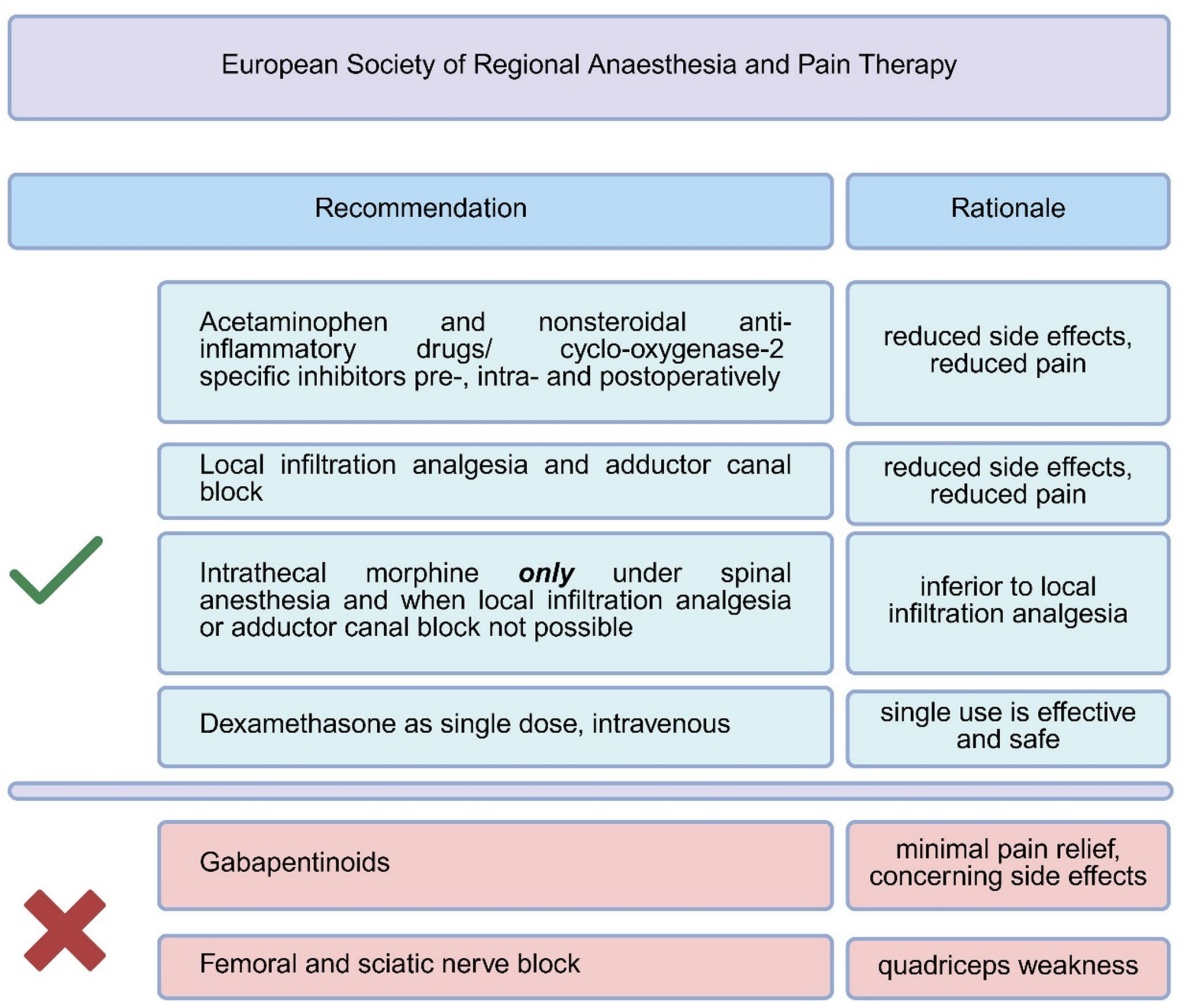
Summary of the pain management options recommended by the European Society of Regional Anaesthesia and Pain Therapy following total knee arthroplasty (Adapted after Lavand’homme et al. [22]) Created by the Authors in BioRender (https://BioRender.com/58obuyw)

In an effort to reduce acute postoperative pain associated with TKA, different regimes of multimodal analgesia have been proposed, which are illustrated in Figs. 1 and 2. However, the large variability and multitude of different treatment protocols and the interpretation of data in the whole context of total joint arthroplasty without a specific focus on TKA makes it difficult for the knee surgeon to identify the most appropriate and effective treatment strategy for pain after TKA.

The aim of this systematic review was to evaluate the efficiency of available analgetic regimes following TKA in reducing postoperative pain and cumulative opioid consumption as rescue medication in a clinically significant manner. However, the large heterogeneity of the published data reporting outcomes of different treatments with various comparators predisposes to an increased risk of bias and therefore, a meta-analysis was not further performed.

## Materials and methods

### Algorithm of the systematic search and selection of studies

A systematic literature search was performed according to the Preferred Reporting Items for Systematic Reviews and Meta-analysis (PRISMA) [23]. The research protocol was registered and approved by the International Prospective Register for Systematic Reviews and Meta-analysis (PROSPERO) under the number CRD42024585740. The systematic database search was performed on PubMed, Embase and Scopus and included combinations of the keywords “duloxetine”, “acetaminophen”, “pregabalin”, “gabapentin”, “steroid”, “opioid”, “NSAID” and “total knee arthroplasty” (Additional Information 1). All available original research articles focusing on different analgesic strategies for patients receiving TKA, published between January 2024 – February 2025, have been further screened based on eligibility criteria.

The inclusion criteria were: randomized-controlled trials (RCTs) and cohort studies written in English and with a minimum level of evidence IV, studies investigating different oral or intravenous analgesic strategies in populations of patients receiving TKA, including bilateral cases, and studies having as primary outcome the quantification of postoperative pain scores or opioid consumption. The exclusion criteria were: studies reporting mixed populations of total hip and knee arthroplasty or unicompartmental knee arthroplasty, studies reporting pain scores only as secondary outcomes or investigating the efficiency of solely nerve blocks with various pharmacologic agents. Published material such as case reports, review articles, experimental studies or non-English articles were also excluded.

### Quality assessment

The quality of the included RCTs was evaluated based on the Jadad Scale, which allows a maximum score of 5 and addresses the quality of randomization, double-blinding and report of loss of follow-up or withdrawal [24]. Studies with minimum score of 3 on the Jadad Scale were considered of good quality and were included in the further analysis. The other clinical studies were evaluated based on the Methodological Index for Non-Randomized Studies (MINORS) for clinical intervention studies, where a maximum score of 24 can be achieved for comparative studies [25]. Papers reporting a MINORS score of minimum 16/24 were included, in order to increase the quality of the review.

### Data extraction and statistical analysis

Author names, year of publication, study design, number of participants, type of anesthesia, periarticular and preemptive analgesia, clinical outcomes such as reported pain levels using Visual Analog scale (VAS) or Numeric Pain Rating scale (NRS) and rescue analgesics consumption were extracted into a Microsoft Excel spreadsheet (MS Microsoft, USA). Continuous variables were presented as medians with interquartile ranges or mean ± standard deviation. A value of *p* < 0.05 was considered statistically significant. Based on the systematic review conducted by Laaigard et al. [26], the minimal clinically important difference (MCID) regarding pain reduction after knee or hip arthroplasty was considered 15/100 mm on VAS at rest and 18/100 mm during movement, respectively, with a relative reduction of 30%. MCID corresponding to consumption of rescue analgesics was set at an absolute difference of 10 mg morphine equivalents i.v. or a relative reduction of 40% [26]. In studies which provided distinct MCID values based on previous personal research, the given MCID was used to evaluate the clinical efficiency.

## Results

**Fig. 3:**
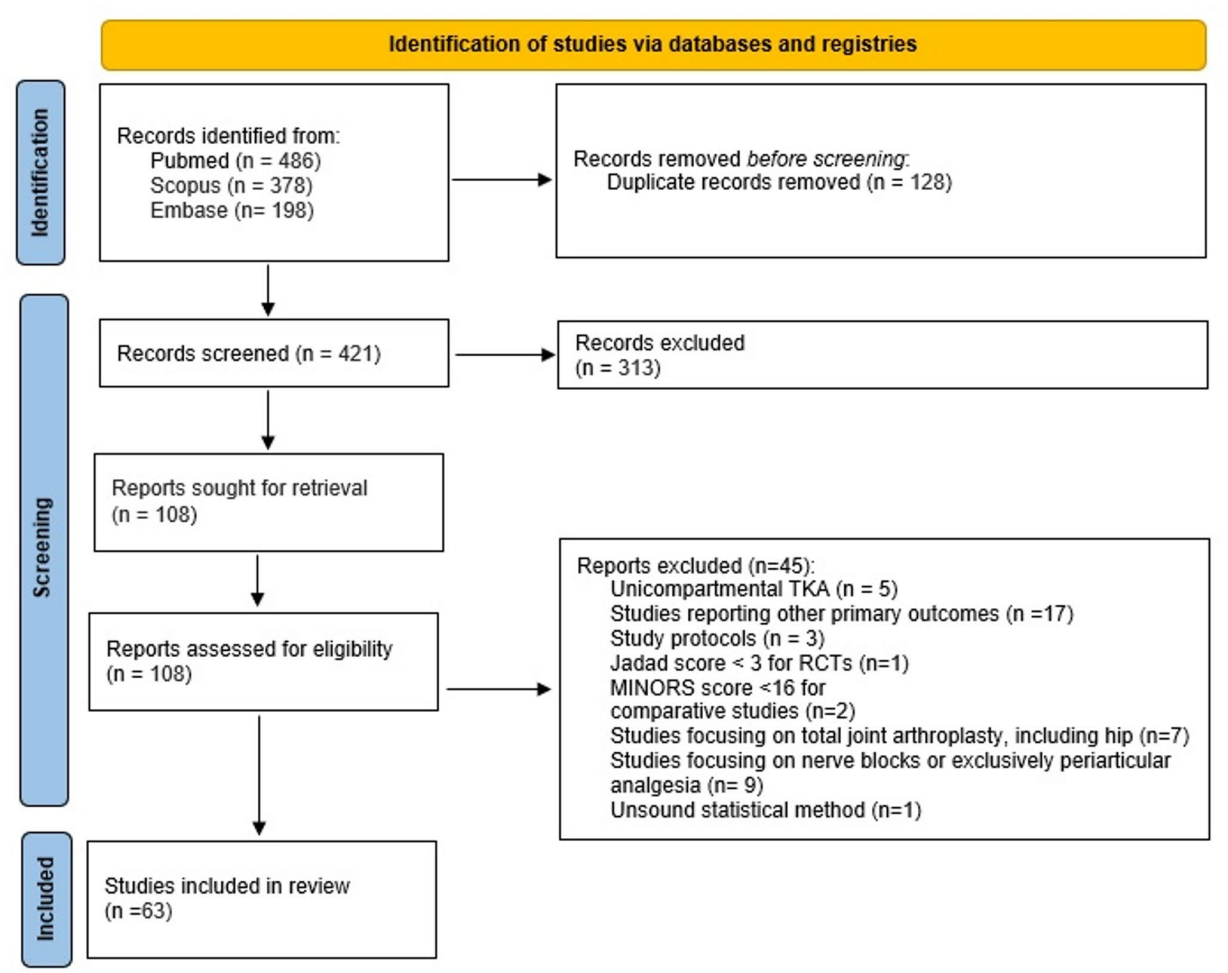
PRISMA diagram illustrating study selection protocol

The initial literature search identified 1062 papers, out of which 63 met all the inclusion criteria according to the selection protocol (Fig. 3).

The median Jadad Scale score for RCTs was 5 (3–5) and the median MINORS for comparative studies was 18 (18-19.5). The presented results included outcomes from 6043 patients enrolled in RCTs and 132 patients enrolled in other types of studies. The identified studies were separately analyzed based on the tested substance class and are presented in detail in Tables 1, 2, 3, 4, 5 and 6.

**Table 1:** Studies reporting clinical outcomes of Duloxetine

No.	Authors, Study type	Patients	Surgical procedure/ Anesthesia	Local infiltration	Basic analgesia	Intervention	Primary outcome and interpretation	Jadad score
1	Rajani, 2024 (RCT) [27]	114	Bilateral TKA in spinal anesthesia	ACB	etoricoxib 60 mg p.o. q.d.	duloxetine 20 mg p.o. q.d. 2 days preoperatively and 28 days postoperatively vs. placebo	rVAS and mVAS (max. 10) rVAS duloxetine vs. placebo at 48 h: 6.38 ± 1.32 vs. 7.02 ± 0.99 (*p* = 0.017, MCID +) at 1 week: 5.83 ± 1.11 vs. 6.82 ± 0.92 (*p* < 0.001, MCID -) at 2 weeks: 3.70 ± 0.89 vs. 4.60 ± 1.03 (*p* < 0.001, MCID -) mVAS duloxetine vs. placebo at 48 h: 7.23 ± 1.12 vs. 8.21 ± 0.69 (*p* < 0.001, MCID -) at 1week: 5.83 ± 1.11 vs. 6.82 ± 0.92 (*p* < 0.001, MCID -) at 2 weeks: 3.70 ± 0.89 vs. 4.60 ± 1.03 (*p* < 0.001, MCID -) No statistically significant difference in rVAS and mVAS between the two groups at 4 weeks and 3 months.	5
2	YaDeau, 2022 (RCT) [28]	160	Unilateral TKA in spinal anesthesia	Spinal-epidural; ACB, iPACK; periarticular injection with bupivacaine, epinephrine and methylprednisolone	APAP 1 g i.v. q.i.d. followed by APAP 1 g p.o. t.i.d.; 15 mg ketorolac i.v. followed by 15 mg meloxicam p.o. q.d.	duloxetine 60 mg p.o. q.d. until POD 14 vs. placebo	NRS duloxetine vs. placebo on POD 1: 5.1 ± 2.6 vs. 5.0 ± 2.6 (non-inferiority *p* < 0.001) on POD 2: 6.2 ± 2.4 vs. 5.8 ± 2.3 (non-inferiority *p* < 0.001) on POD 14:4.2 ± 2.0 vs. 4.8 ± 2.2 (non-inferiority *p* = 0.002) Cumulative opioid consumption POD 1–14 duloxetine vs. placebo: 288 ± 226 OME vs. 432.5 ± 374 OME (*p* = 0.003, MCID +)	5
3	Yuan, 2022 (RCT) [29]	100	Unilateral TKA in general anesthesia	Periarticular injection with ropivacaine	Preemptive analgesia with celecoxib	duloxetine 60 mg p.o. q.d. 2 days preoperatively – POD 14 vs. placebo	rVAS and mVAS (max. 10) rVAS duloxetine vs. placebo at 24 h: 4.7 ± 2.3 vs. 5.9 ± 2.6 (*p* = 0.016, MCID -) at 7 days: 2.1 ± 1.6 vs. 2.8 ± 1.7 (*p* = 0.037, MCID -) mVAS duloxetine vs. placebo at 24 h: 6.2 ± 2.1 vs. 7.1 ± 2.2 (*p* = 0.039, MCID -) at 7 days: 3.3 ± 1.7 vs. 4.1 ± 2.0 (*p* = 0.034, MCID -)	5
4	YaDeau, 2016 (RCT) [30]	106	Unilateral TKA in spinal anesthesia	Epidural analgesia and ACB	meloxicam 15 mg p.o. q.d.	duloxetine 60 mg p.o. q.d. until POD 15 vs. placebo	NRS duloxetine vs. placebo on POD 14: 3.5 ± 2.1 vs. 3.8 ± 2.3 (*p* = 0.386)	5
5	Ho, 2010 (RCT) [31]	50	Unilateral TKA in spinal anesthesia	n. m.	APAP 1 g i.v. q.i.d.	duloxetine 60 mg 2 doses p.o. (one preoperatively and one on POD 1) vs. placebo	Cumulative consumption of morphine i.v. in duloxetine vs. placebo group at 48 h: 19.5 ± 14.5 vs. 30.3 ± 18.1 mg (*p* = 0.017, MCID +)	5
6	Kim, 2021 (RCT) [32]	39	Unilateral TKA in general anesthesia	Periarticular injection with ropivacaine, morphine and ketorolac, no postoperative nerve block	Preemptive analgesia with celecoxib 200 mg and pregabalin 150 mg. + oxycodone 10 mg and celecoxib 200 mg p.o. q.d., tramadol 37.5 mg and APAP 650 mg p.o. b.i.d. for 7 days	duloxetine 30 mg p.o. q.d. 14 days preoperatively and 8 weeks postoperatively vs. placebo	rVAS and mVAS (max. 10) rVAS duloxetine vs. placebo on POD 1: 3.5 ± 1.4 vs. 5.7 ± 1.4 (*p* < 0.001, MCID +) at 1 week: 2.5 ± 1.3 vs. 4.3 ± 1.6 (*p* < 0.001, MCID +) at 6 weeks: 1.9 ± 1.2 vs. 3.3 ± 1.7 (*p* = 0.014, MCID -) at 12 weeks: 2.2 ± 1.3 vs. 2.8 ± 1.0 (*p* = 0.205) mVAS duloxetine vs. placebo on POD 1: 5.0 ± 1.6 vs. 7.3 ± 1.4 (*p* < 0.001, MCID +) at 1 week: 4.1 ± 1.2 vs. 6.0 ± 1.6 (*p* < 0.001, MCID +) at 6 weeks: 2.4 ± 1.6 vs. 4.0 ± 1.7 (*p* = 0.011, MCID -) at 12 weeks: 2.6 ± 1.3 vs. 3.4 ± 0.8 (*p* = 0.068)	5
7	Kim, 2021 (RCT) [33]	239	Unilateral TKA in general anesthesia	Preemptive analgesia with celecoxib 200 mg and pregabalin 150 mg, no postoperative nerve block	celecoxib 200 mg p.o. q.d. and APAP 650 mg p.o. b.i.d. for 6 weeks	oxycodone-naloxone 10/5 mg p.o. b.i.d. vs. duloxetine 30 mg p.o. q.d. 6 weeks postoperatively	mVAS (max. 10) mVAS duloxetine vs. opioid group at 6 weeks: 3.4 ± 1.5 vs. 3.5 ± 1.6 (*p* = 0.43) at 3 months: 2.7 ± 1.5 vs. 2.8 ± 1.4 (*p* = 0.72) at 6 months: 2.2 ± 0.8 vs. 2.2 ± 0.9 (*p* = 0.245)	3
8	Imani, 2023 (RCT) [34]	60	Unilateral TKA in spinal anesthesia	n. m.	n.m.	pregabalin 75 p.o. vs. duloxetine 30 mg p.o. vs. placebo 90 min before surgery, then 12 and 24 h postoperatively	VAS at 48 h (max. 10) VAS at 48 h pregabalin vs. duloxetine vs. placebo: 2.1 ± 0.9 vs. 1.9 ± 0.8 vs. 2.7 ± 1.0 (*p* = 0.01, between pregabalin and duloxetine not significant)	5
9	Pinsornsak, 2024 (RCT) [35]	84	Unilateral TKA in spinal anesthesia	bupivacaine, adrenaline, ketorolac, morphine	celecoxib 200 mg p.o. q.d. and APAP 1000 mg p.o. t.i.d.	duloxetine 30 mg p.o. q.d. in the study group for 6 postoperative weeks	VAS (max. 10) for pain at rest, during walking, and at night at 24 h, 72 h, 2 weeks, 6 weeks, and 12 weeks (MCID = 2.26) No difference between groups except night pain at 2 weeks, which was significantly lower in the duloxetine group (1.0 ± 0.9 vs. 2.0 ± 1.5, *p* < 0.001, MCID -)	4

*ACB * adductor canal block, *APAP*  cetaminophen, *b.i.d.* twice daily, *i.v.* intravenous, *iPACK* nfiltration between popliteal artery and capsule of the knee, *MCID* minimal clinically important difference, *mVAS* pain on visual analog scale during motion, *n.m.* not mentioned, *NRS* numeric rating scale, *OME*   oral morphine equivalents,

*p.o.* per os, *POD * postoperative day, *q.d.* once daily, *RCT* randomized controlled trial, *rVAS* pain on visual analog scale at rest, *TKA* total knee arthroplasty

**Table 2:** Studies reporting clinical outcomes of acetaminophen

No.	Authors, Study type	Patients	Surgical procedure/ Anesthesia	Local infiltration	Basic analgesia	Intervention	Primary outcome and interpretation	Jadad score
1	Seki, 2023 (RCT) [36]	98	Unilateral TKA in general anesthesia	Single-shot FNB and periarticular infiltration with levobupivacaine	celecoxib 400 mg p.o. q.d.+ APAP 3000 mg/day p.o.	APAP 1 g i.v. q.i.d. vs. placebo on the day of surgery	Postoperative NRS at rest NRS APAP i.v. vs. control at 48 h: 3.0 ± 1.7 vs. 4.0 ± 2.3 (*p* = 0.034, MCID -)	3
2	Wang, 2023 (RCT) [37]	80	Unilateral TKA in general anesthesia	ACB, periarticular infiltration with ropivacaine and epinephrine	celecoxib 400 mg + pregabalin 150 mg p.o. preemptive	APAP 300 mg vs. placebo p.o. 2 h before surgery and then b.i.d,	Cumulative consumption of morphine s.c. APAP vs. placebo at 24 h: 11.3 ± 6.5 mg vs. 12.3 ± 7.7 mg (*p* = 0.445)	4
3	O’Neal, 2017 (RCT) [38]	174	Unilateral TKA in spinal anesthesia	Peri-capsular injection with ropivacaine, ketorolac, clonidine and epinephrine	Standard preoperative celecoxib and oxycodone	APAP 1 g i.v. and placebo p.o. vs. APAP 1 g p.o. and placebo i.v. after surgery vs. placebo p.o. and i.v.	average NRS in the post-anesthesia care unit: 0.56 ± 0.99 in the i.v. group vs. 0.67 ± 1.20 in the p.o. group vs. 0.58 ± 0.99 in the placebo group (*p* = 0.71)	5
4	Park, 2015 (RCT) [39]	320	Unilateral TKA, anesthesia n.m.	n.m.	N/A	tramadol/APAP-extended release (TA-ER) 75/650 mg p.o. vs. tramadol/APAP-immediate release (TA-IR) 37.5/325 mg p.o.	difference in sum of pain intensity difference (SPID) from the time of qualification (POD1) to final assessment at 48 h (POD 3) with non-inferiority of TA-ER to TA-IR demonstrated by post-hoc analysis	5
5	Richards 2013 (RCT) [40]	40	Unilateral TKA in general or spinal anesthesia	n.m.	N/A	flexible dose regimen of morphine/ oxycodone (3 mg/2 mg to 24 mg/16 mg) vs. fixed low-dose morphine/oxycodone regimen (3 mg/2 mg) vs. oxycodone/ APAP (5 mg/325 mg	Timeweighted sum of pain intensity differences (SPID) by using NRS from 0 to 48 h after the first dose of analgesia (SPID48). None of the pairwise comparisons showed a significant difference in SPID48 values.	3
6	Murata-Ooiwa, 2017 (RCT) [41]	67	Unilateral TKA in spinal anesthesia	Periarticular injection with methylprednisolone, ropivacaine, morphine, epinephrine and ketoprofen.	loxoprofen 60 mg p.o. t.i.d.	APAP 1 g i.v. t.i.d. until POD 2, then q.d. until POD 5 vs. placebo	Postoperative rVAS (max. 100 mm) rVAS APAP vs. placebo on POD 1: 15.3 ± 17.0 vs. 26.8 ± 19.0 (*p* = 0.013, MCID -)	5
7	Lubis, 2021 (RCT) [42]	36	Unilateral TKA in spinal anesthesia	n.m.	n.m.	Group 1 (APAP 1 g i.v. + ibuprofen 800 mg i.v. at the end of the surgery, and then q.i.d. up to 24 h) vs. Group 2 (APAP 1 g i.v. at the end of the surgery, and then q.i.d. up to 24 h) vs. Group 3 (ibuprofen 800 mg i.v. at the end of surgery, and then q.i.d. up to 24 h).	Cumulative morphine consumption at 24 h Group 1 vs. Group 2 vs. Group 3: 7.5 mg (30–36.0) vs. 15.0 mg (4.5–28.5) vs. 9.0 mg (0.0–24.0) (*p* = 0.391)	3

*ACB* adductor canal block, *APAP* acetaminophen, *b.i.d.* twice daily, *FNB* femoral nerve block, *i.v.* intravenous, *MCID* minimal clinically important difference, *n.m.* not mentioned, *N/A *  not applicable, *NRS* numeric rating scale, *p.o.* per os, *POD* postoperative day, *q.d.* once daily, *q.i.d.* four times daily, *RCT* randomized controlled trial, *rVAS* pain on visual analog scale at rest, *t.i.d.* three times daily, *TKA*  total knee arthroplasty

**Table 3:** Studies reporting clinical outcomes of pregabalin, Gabapentin and nefopam

No.	Authors, Study type	Patients	Surgical procedure/ Anesthesia	Local infiltration	Basic analgesia	Intervention	Primary outcome and interpretation	Jadad score
1	Zhou, 2023 (RCT) [43]	160	Unilateral TKA in general anesthesia	Periarticular infiltration with ropivacaine, triamcinolone, epinephrine. No nerve block	N/A	Pregabalin 150 mg + placebo p.o. vs. celecoxib 200 mg + placebo p.o. vs. pregabalin 150 mg + celecoxib 200 mg p.o. vs. placebo p.o. 12 h and 2 h prior to surgery	Cumulative sufentanil consumption at 48 h (MCID: reduction of 30%): Placebo 74.1 ± 15.2 µg Celecoxib 62.3 ± 12.9 µg Pregabalin 62.1 ± 13.5 µg Celecoxib + Pregabalin 49.3 ± 10.9 µg (*p* = 0.0003, statistically significant between Celecoxib + Pregabalin vs. all other groups)	5
2	YaDeau, 2015 (RCT) [44]	120	Unilateral TKA in spinal anesthesia	FNB with bupivacaine and epinephrine	meloxicam 15 mg p.o. q.d.	Pregabalin 0 mg, 50 mg, 100 mg, 150 mg p.o. 30 min before surgery and then b.i.d. for 14 days	NRS pain score with flexion on POD 14 Pregabalin 0 mg: 4.0 ± 2.3 Pregabalin 50 mg: 4.8 ± 2.1 Pregabalin 100 mg: 4.5 ± 2.4 Pregabalin 150 mg: 4.0 ± 2.1 (*p* = 0.55 per intention-to-treat)	5
3	Lee, 2015 (RCT) [45]	41	Unilateral TKA in general anesthesia	Periarticular injection with bupivacaine, morphine, epinephrine, methylprednisolone	PCA with fentanyl	celecoxib 400 mg p.o.+ pregabalin 150 mg p.o. vs. celecoxib 400 mg p.o.	Fentanyl consumption at 48 h (MCID = 80 µg at 6 h, 40% reduction) pregabalin + celecoxib vs. celecoxib: 701.9 ± 126.32 µg vs. 857.0 ± 232.06 µg (*p* = 0.01, MCID-) NRS pregabalin + celecoxib vs. celecoxib at rest post-TKA 6 h: 2.6 ± 0.7 vs. 3.5 ± 1.5 (*p* = 0.02) 12 h: 2.5 ± 1.0 vs. 3.5 ± 1.3 (*p* = 0.01) 24 h: 2.7 ± 1.1 vs. 3.4 ± 1.3 (*p* = 0.11) 48 h: 2.8 ± 1.2 vs. 3.3 ± 1.2 (*p* = 0.21) NRS pregabalin + celecoxib vs. celecoxib on maximal flexion post-TKA 6 h: 3.2 ± 0.8 vs.4.4 ± 1.3 (*p* = 0.002) 12 h: 3.1 ± 1.1 vs.4.6 ± 1.3 (*p* = 0.001) 24 h: 3.4 ± 1.0 vs. 4.3 ± 1.4 (*p* = 0.03) 48 h: 3.3 ± 0.9 vs. 4.2 ± 1.3 (*p* = 0.01)	3
4	Buvanendran, 2010 (RCT) [46]	240	Unilateral TKA in spinal anesthesia	Local infiltration with bupivacaine and epinephrine	celecoxib 400 mg p.o. 1–2 before surgery, then 200 mg p.o. b.i.d. until POD 3	Pregabalin 300 mg p.o. 1–2 h before surgery, then pregabalin p.o. b.i.d. 150 mg until POD 10, 75 mg on POD 11–12, 50 mg on POD 13–14 vs. placebo	Lower extremity neuropathic pain at 3 and 6 months after TKA using a measure administered during a telephone interview (MCID = 75% reduction in the incidence of neuropathic pain at 6 months) 3 months: neuropathic pain incidence after TKA 0% in the pregabalin group vs. 8.7% in the placebo group (*p* = 0.001, MCID +). 6 months: neuropathic pain incidence after TKA 0% in the pregabalin group vs. 5.2% in the placebo group (*p* = 0.014, MCID +).	5
5	Yik, 2019 (RCT) [47]	87	Unilateral TKA in general anesthesia	FNB with ropivacaine	APAP 1 g p.o. q.i.d. + etoricoxib 120 mg p.o. q.d. for 72 h postoperati-vely	Pregabalin 75 mg p.o. 1 h prior to surgery, followed by another 75 mg p.o. q.d. for 48 h postoperatively vs. placebo	Cumulative morphine i.v. consumption at 72 h post-TKA (MCID = 50% reduction) pregabalin vs. placebo: 14.0 mg (0.0–144.0) vs. 14.0 (1.0–81.0) (p n.s.)	5
6	Pinsornsak, 2022 (RCT) [48]	84	Unilateral TKA in spinal anesthesia	ACB and local infiltration with bupivacaine, adrenaline, morphine, ketorolac	naproxen 250 mg p.o. b.i.d. + APAP 1 g p.o. q.i.d. + gabapentin 300 mg p.o. q.d. for 48 h	Continuous i.v. infusion of nefopam 80 mg in 5% dextrose in water 500 mL for 24 h vs. 5% dextrose in water 500 mL.	rVAS and VAS during knee motion (max. 100 mm) at 6, 12, 18, 24, 30, 36, 42, and 48 h post-TKA rVAS nefopam vs. placebo at 6 h: 20 ± 27 mm vs. 36 ± 24 mm (*p* = 0.01, MCID +) rVAS nefopam vs. placebo at 12–48 h: p n.s. VAS during knee flexion nefopam vs. placebo at 6–48 h: p n.s.	5
7	Lunn, 2015 (RCT) [49]	300	Unilateral TKA	n.m.	Standardized multimodal analgesic regime	Group A (gabapentin 1300 mg) vs. Group B (gabapentin 900 mg) vs. Group C (placebo) daily from 2 h preoperatively to POD 6	mVAS (max. 100 mm) at 24 h post-TKA A vs. B. vs. C: 41 mm (37–46) vs. 41 mm (36–45) vs. 42 mm (37–47) (*p* = 0.93)	5
8	Petersen, 2018 (RCT) [50]	215	Unilateral TKA	n.m.	Standardized multimodal analgesic regimen	Gabapentin 1300 mg vs. gabapentin 900 mg vs. placebo daily from 2 h preoperatively to POD 6	Pain intensity scores during walking, at rest (supine), upon 45 ° hip flexion with straight leg, and upon passive 60 ° knee flexion after 3–4 years post-TKA Gabapentin does not influence the development of chronic postoperative pain or the revision rate at 3–4 year-follow-up after TKA.	5
9	Wang, 2024 (RCT) [51]	100	Unilateral TKA in general anesthesia	ACB and local infiltration with ropivacaine, epinephrine and dexamethasone	Celecoxib 200 mg p.o. b.i.d. + pregabalin 150 mg p.o. b.i.d.	nefopam 40 mg b.i.d. vs. placebo	Cumulative consumption of opioids post-TKA Oxycodone p.o. consumption in nefopam vs. placebo group At 24 h: 13.6 ± 8.0 mg vs. 22.2 ± 8.9 mg (*p* < 0.001, MCID +) During hospitalization: 20.2 ± 13.2 mg vs. 30.4 ± 13.5 mg (*p* < 0.00, MCID -) Morphine i.v. consumption at 24 h nefopam vs. placebo At 24 h: 3.4 ± 5.2 mg vs. 6.4 ± 6.0 mg (*p* = 0.0089, MCID +) During hospitalization: 4.2 ± 5.4 mg vs. 8.8 ± 7.2 mg (*p* = 0.001, MCID +) Reported differences may not be clinically relevant	3

*ACB* adductor canal block, *APAP* acetaminophen, *b.i.d. * twice daily, *FNB* femoral nerve block, *i.v.* intravenous, *MCID* minimal clinically important difference, *mVAS* pain on visual analog scale during motion, *n.m.* not mentioned, *n.s.* not significant, *N/A* not applicable, *NRS* numeric rating scale, *p.o.* per os, *PCA* patient-controlled analgesia, *POD* postoperative day, *q.d.* once daily, *q.i.d.* four times daily, *RCT*  andomized controlled trial, *rVAS* pain on visual analog scale at rest, *TKA * total knee arthroplasty.

**Table 4:** Studies reporting clinical outcomes of opioid analgesics

No.	Authors, Study type	Patients	Surgical procedure/ Anesthesia	Local infiltration	Basic analgesia	Intervention	Primary outcome and interpretation	Jadad score
1	Wang, 2023 (RCT) [52]	100	Unilateral TKA in general anesthesia	Ropivacaine and epinephrine	N/A	2 h before surgery celecoxib 400 mg p.o., pregabalin 150 mg p.o. and extended-release oxycodone 10 mg p.o. vs. celecoxib 400 mg p.o., pregabalin 150 mg p.o. and placebo.	postoperative consumption of morphine s.c. preemptive opioid group vs. placebo: at 24 h: 12.4 ± 7.2 mg vs. 11.4 ± 8.1 mg (*p* = 0.41) total consumption: 19.8 ± 8.9 mg vs. 18.2 ± 12.4 mg (*p* = 0 0.22) Preemptive opioid administration did not provide clinical benefits over placebo	5
2	Haffar, 2022 (RCT) [53]	80	Unilateral TKA in spinal anesthesia	ACB with ropivacaine, no intraarticular cocktail	APAP 1 g p.o. q.i.d., gabapentin 300 mg p.o. t.i.d., meloxicam 15 mg p.o. q.d.	Topical Cannabidiol (CBD) vs. essential oil (EO) vs. CBD + EO vs. placebo t.i.d. until POD 14	VAS on POD 0, 1, 2, 7, 14, 42 (max. 100 mm, MCID = 22.4) CBD vs. EO vs. CBD + EO vs. placebo: 69.9 ± 19.3 mm vs. 51.0 ± 18.2 mm vs. 61.0 ± 20.7 mm vs. 61.4 ± 15.1 mm (*p* = 0.026, MCID -) No significant differences VAS pain scores on POD 0, 1,7,14,42	5
3	Hall, 2015 (RCT) [54]	38	Unilateral TKA, anesthesia n.m.	n.m.	n.m.	morphine 1 mg bolus vs. fentanyl patch 12.5 µg (> 65 years) or 25 mcg (< 65 years)	VAS pain score on POD 5 No significant difference in rVAS, mVAS and worst pain between morphine and fentanyl	3
4	Kuusniemi, 2012 (RCT) [55]	137	Unilateral TKA, anesthesia n.m.	Epidural analgesia for 48 h	N/A	Oxycodone-naloxone prolonged release (20/10 mg < 65years or 10/5 mg > 65 years) (OXN PR) vs. oxycodone prolonged release (20 mg < 65 years or 10 mg > 65 years) (OXY PR) POD 1–3	24-h average pain intensity score at rest No clinically relevant differences in the 24-h average pain intensity score at rest in the per protocol population (not analyzed statistically). Non-inferiority of OXN PR compared with OXY PR.	5
5	Wang, 2020 (RCT) [56]	100	Unilateral TKA in general anesthesia	Detailed in Intervention	Preemptive analgesia with celecoxib 200 mg	Periarticular infiltration with ropivacaine, epinephrine, and morphine vs. ropivacaine and epinephrine	postoperative total consumption of morphine i.v. (MCID = 30% reduction) morphine intraarticular group vs. control: 12.6 ± 8.3 mg vs. 18.8 ± 7.7 mg (*p* < 0.001, MCID +) No significant difference between the two groups in postoperative rVAS and mVAS.	5
6	Olivella, 2023 (RCT) [57]	81	Unilateral TKA	Local periarticular infiltration with morphine, ketorolac and bupivacaine	N/A	Group 1: ketorolac 30 mg i.v. q.i.d. <65 years or 15 mg > 65 years + APAP 1 g i.v. q.i.d. Group 2: morphine 0.1 mg/kg i.v. q.i.d. and oxycodone/APAP (5/650 mg) p.o. q.i.d.	NRS score post-TKA Group 1 vs. Group 2 at 12 h: 6.7 ± 2.9 vs. 5.9 ± 2.8 (*p* = 0.209) at 24 h: 6.2 ± 2.0 vs. 6.1 ± 2.2 (*p* = 0.813) at 48 h: 4.7 ± 2.1 vs. 4.6 ± 1.7 (*p* = 0.835)	3
7	Iwakiri, 2017 (RCT) [58]	102	Unilateral TKA in general anesthesia	No postoperative anesthetic blocks	celecoxib for 21 days	Periarticular intraoperative injection with ropivacaine, epinephrine, ketoprofen, methylprednisolone ± morphine 10 mg	mVAS morphine group vs. control (max. 100 mm, MCID = 20 mm) POD 1: 21.5 ± 25.9 vs. 19.1 ± 24.4 (*p* = 0.81) POD 7: 30.9 ± 25.2 vs. 25.3 ± 25.6 (*p* = 0.4) POD 14: 17.3 ± 21.4 vs. 15.2 ± 16.5 (*p* = 0.6) POD 21: 10.8 ± 17.2 vs. 10.0 ± 14.5 (*p* = 0.86)	5
8	Manassero, 2018 (RCT) [59]	112	Unilateral TKA in spinal anesthesia	Sciatic and femoral nerve block with ropivacaine	N/A	OXN group (prolonged-release oxycodone/ naloxone 25/12.5 mg p.o. daily) vs. Control group (morphine 2 mg bolus administered via PCA)	NRS pain scores at rest and during movement during the first 48 h post-TKA (MCID not specified) OXN group vs. Control group at rest at 0–24 h: 0.89 ± 1.54 vs. 1.27 ± 1.82 (*p* = 0.0019) at 25–48 h: 1.03 ± 1.69 vs. 1.65 ± 2.05 (*p* = 0.0006) No statistically significant difference in NRS pain score during movement between the two groups.	3

*ACB * adductor canal block, *APAP* acetaminophen, *i.v.* intravenous, *MCID*  minimal clinically important difference, mVAS   pain on visual analog scale during motion, *n.m.* not mentioned, *N/A* not applicable, *NRS *  numeric rating scale, *p.o.* per os, *PCA * patient-controlled analgesia, *POD* postoperative day, *q.d.* once daily, *q.i.d.* four times daily, *RCT* randomized controlled trial, r*VAS *  pain on visual analog scale at rest, *s.c.* subcutaneous, *t.i.d.* three times daily, *TKA* total knee arthroplasty.

**Table 5:** Studies reporting clinical outcomes of steroids

No.	Authors, Study type	Patients	Surgical procedure/ Anesthesia	Local infiltration	Basic analgesia	Intervention	Primary outcome and interpretation	Jadad score
1	Wu, 2023 (RCT) [60]	90	Unilateral TKA in general anesthesia	Periarticular infiltration with ropivacaine, epinephrine	celecoxib 200 mg p.o. b.i.d. + oxycodone 10 mg p.o. b.i.d.	dexamethasone 10 mg periarticular + dexamethasone 10 mg i.v. vs. placebo	rVAS and VAS during flexion post-TKA (max. 100 mm, MCID = 10 mm) rVAS dexamethasone vs. control at 6 h: 32.7 ± 8.1 vs. 39.4 ± 7.2 (*p* < 0.001, MCID -) at 12 h: 37.0 ± 7.2 vs. 41.7 ± 8.1 (*p* = 0.004, MCID -) at 24 h: 37.5 ± 7.0 vs. 44.1 ± 5.8 (*p* < 0.001, MCID -) No significant differences at 2 h, 48 h, 3 months. VAS during flexion dexamethasone vs. control at 2 h: 39.7 ± 8.3 vs. 46.5 ± 9.7 (*p* = 0.001, MCID -) at 6 h: 45.8 ± 8.6 vs. 57.4 ± 9.2 (*p* < 0.001, MCID +) at 12 h: 51.1 ± 9.1 vs. 69.3 ± 7.1 (*p* < 0.001, MCID +) at 24 h: 53.1 ± 8.6 vs. 71.3 ± 7.4 (*p* < 0.001, MCID +) No significant differences at 48 h and 3 months.	5
2	Shaw, 2023 (RCT) [61]	109	Unilateral TKA in spinal anesthesia	n.m.	NSAIDs, APAP	dexamethasone 4 mg p.o. b.i.d. on POD 1–4 vs. placebo	Postoperative VAS score Significantly lower average VAS in the first 4 days in the dexamethasone group vs. placebo (3.77 vs. 4.52 cm, *p* = 0.01).	5
3	Nielsen, 2023 (RCT) [62]	160	Unilateral TKA in spinal anesthesia	Local infiltration with ropivacaine, no peripheral nerve blocks	APAP 1 g p.o. q.i.d. + celecoxib 200 mg p.o. b.i.d.	1 dose dexamethasone 1 mg/kg (HD) vs. 0.3 mg/kg i.v. (ID)	Percentage of subjects experiencing moderate to severe pain (VAS > 30 mm) upon walking 5 m at 24 h similar percentages of patients experiencing moderate to severe pain after 24 h in the two groups (56% HD vs. 53% ID, *p* = 0.65)	5
4	Saini, 2023 (RCT) [63]	180	Unilateral TKA in spinal anesthesia	Periarticular injection with ropivacaine, fentanyl, adrenaline, clonidine, ketorolac	diclofenac 75 mg i.v. t.i.d. + APAP 650 mg p.o. q.i.d. + pregabalin 75 mg p.o.	Periarticular dexamethasone 8 mg (PAID) vs. i.v. dexamethasone 8 mg before anesthesia (SDIV) vs. i.v. 4 mg dexamethasone before the anesthesia and i.v. 4 mg 24 h later (LID)	VAS pain score at 24 h and 48 h (max. 100 mm, MCID not defined) Mean differences in VAS scores between groups at 24 h PAID vs. LID: -10.58, *p* = 0.0005 at 24 h SDIV vs. LID: -9.16, *p* = 0.0005 at 48 h PAID vs. LID: -9.00, *p* = 0.0005 at 48 h SDIV vs. LID: -7.41, *p* = 0.0005 No difference between PAID and SDIV at 24 and 48 h.	5
5	Gasbjerg, 2022 (RCT) [64]	485	Unilateral TKA in spinal or general anesthesia	Local infiltration analgesia with ropivacaine	APAP 1 g p.o. and ibuprofen 400 mg p.o. given 1 h before, then q.i.d.	DX1 (dexamethasone 24 mg i.v. before anesthesia + placebo 24 h later) DX2 (dexamethasone 24 mg i.v. + dexamethasone 24 mg i.v. 24 h later) vs. placebo (placebo + placebo)	Total opioid consumption in morphine i.v. equivalents at 48 h post-TKA (MCID = 10 mg morphine i.v.) DX1 vs. DX 2 vs. placebo: 37.9 mg (20.7–56.7) vs. 35.0 mg (20.6–52.0) vs. 43.0 mg (28.7–64.0) difference DX1 – placebo: 7.8 mg, *p* = 0.008 (MCID -) difference DX2 – placebo: 10.7 mg, *p* < 0.001 (MCID +)	5
6	Nielsen, 2022 (RCT) [65]	88	Unilateral TKA in spinal anesthesia	Local infiltration with ropivacaine, no peripheral nerve blocks	APAP 1 g p.o. q.i.d. + celecoxib 200 mg p.o. b.i.d.	One administration dexamethasone 1 mg/kg (HD) vs. 0.3 mg/kg i.v (ID)	Percentage of subjects experiencing moderate to severe pain (VAS > 30 mm) upon walking 5 m at 24 h (MCID = reduction of 50%). lower percentage of subjects reporting moderate-to-severe pain at 24 h in the HD group compared with the ID group in a 5 m walk test (49% HD vs. 79% ID, *p* < 0.01, MCID -).	5
7	Cheng, 2021 (RCT) [66]	98	Unilateral TKA in spinal anesthesia	Local infiltration with epinephrine, betamethasone, ropivacaine and morphine	celecoxib 200 mg p.o. b.i.d. and tramadol 100 mg p.o. q.d.	Prednisone 10 mg p.o. q.d. on POD 1–14 vs. control	rVAS and mVAS (max. 10 mm, MCID = 2.5 mm reduction of mVAS on POD 14) Prednisone group vs. Control mVAS preoperative: 4.9 ± 1.3 vs. 4.8 ± 1.2 (*p* > 0.05) mVAS on POD 14: 2.3 ± 1.0 vs. 4.0 ± 1.5 (*p* < 0.05, MCID +) rVAS preoperative: 3.3 ± 0.7 vs. 3.2 ± 0.8 (*p* > 0.05) rVAS on POD 14: 2.1 ± 0.7 vs. 3.3 ± 1.0 (*p* < 0.05, MCID n.m.)	3
8	Li, 2021 (RCT) [67]	90	Unilateral TKA in general anesthesia	Local infiltration with ropivacaine and epinephrine	loxoprofen p.o. and parecoxib i.m. b.i.d. until discharge	dexamethasone 10 mg i.v. during surgery (DEX) vs. topical infiltration with dexamethasone 0.1 mg/ml (topical DEX)	Postoperative rVAS and mVAS (max. 10 mm, MCID = reduction of 1 mm in VAS) rVAS in DEX vs. topical DEX group at 2 h: 2.7 ± 0.6 vs. 2.3 ± 0.5 (*p* = 0.002, MCID -) at 6 h: 3.0 ± 0.7 vs. 2.6 ± 0.7 (*p* = 0.005, MCID -) at 12 h: 3.5 ± 0.8 vs. 3.1 ± 0.7(*p* = 0.047, MCID -) No differences at 2 h, 24 h, 48 h and 3 months. mVAS in DEX vs. topical DEX group at 12 h: 5.8 ± 1.1 vs. 5.3 ± 0.8 (*p* = 0.015, MCID -) at 24 h: 5.5 ± 1.1 vs. 5.1 ± 0.7 (*p* = 0.043, MCID -) No differences at 2 h, 6 h, 48 h and 3 months. No differences in cumulative morphine consumption between groups.	5
9	Liszka, 2022 (RCT) [68]	160	Unilateral TKA in spinal anesthesia	FNB	Postoperative APAP i.v. max. 4 g/day, or metamizole i.v. max. 5 g/day	Group M: preemptive analgesia with gabapentin 300 mg p.o. + methylprednisolone 125 mg i.v. Group K: preemptive placebo p.o. + placebo i.v.	NRS score at rest Significantly lower NRS pain scores in Group M (study group) vs. Group K (Control) at 6, 12, 18 and 24 h (continuous data not available).	5
10	Li, 2024 (RCT) [69]	163	Unilateral TKA in general anesthesia	ACB, local infiltration with ropivacaine and flurbiprofen	celecoxib 200 mg p.o. b.i.d	dexamethasone 10 mg i.v. (IV) vs. dexamethasone 10 mg periarticular (PI).	rVAS and mVAS at 6 h and on POD 1–4 (max. 10 mm, MCID = 1.3 mm reduction on VAS) Similar rVAS at 6 h and on POD 1–4. mVAS on POD 2 lower in the PI group (IV vs. PI: 2.73 ± 1.69 vs. 2.08 ± 1.45, *p* = 0.039, MCID -) Similar mVAS at 6 h and on POD 1, 3,4.	5
11	Cheng, 2019 (RCT) [70]	60	Unilateral TKA in spinal anesthesia	Infiltration with ropivacaine, ketorolac, adrenaline	APAP, etoricoxib, gabapentin	methylprednisolone 125 mg i.v. before surgery vs. control	VAS at rest, during maximal knee flexion and straight raise with 45° hip flexion and frame walking for 5 m at 24, 30 and 48 h post-TKA All VAS scores significantly lower in the study group compared to control at 24 and 30 h (*p* < 0.05, MCID +, continuous data not available). No differences at 6 and 48 h.	5
12	Chan, 2021 (RCT) [71]	46	Bilateral TKA in spinal anesthesia	No peripheral nerve blocks. Local infiltration with ropivacaine, ketorolac, adrenaline	APAP 1 g, pregabalin 75 mg, celecoxib 200 mg p.o.	dexamethasone 16 mg i.v. before anesthesia in all patients ± 40 mg triamcinolone periarticular in only one knee	Postoperative rVAS and mVAS (max. 10 mm, MCID = 1 mm reduction in VAS) Knees receiving periarticular triamcinolone showed significantly lower mVAS from POD 1 to 6 weeks and significantly lower rVAS on POD 3, 6 and at 6 weeks with the largest reduction at 6 weeks (MCID not met, continuous data not available)	5
13	Kitcharanant2024 (RCT) [72]	49	Unilateral TKA in spinal anesthesia	n.m.	etoricoxib 90 mg p.o., APAP 500 mg p.o. q.i.d.	dexamethasone 10 mg i.v. prior to anesthesia and at 24 h and 48 h postoperative vs. placebo (saline solution)	Modified WOMAC pain score (VAS 0-500) at 12 weeks postoperatively (MCID = 12 points) Dexamethasone vs. Placebo group 9.38 ± 17.77 mm vs. 23.75 ± 23.56 mm (mean difference 7.875, *p* = 0.607)	5
14	Afshar, 2025 (RCT) [73]	90	Unilateral TKA in spinal anesthesia	bupivacaine, no nerve blocks	APAP 1000 mg i.v. q.i.d.	Group A (dexamethasone 4 mg i.v.) vs. Group B (dexamethasone 8 mg i.v.) vs. Group C (dexamethasone 16 mg i.v.) after spinal anesthesia	VAS pain score (max. 100 mm) at 1, 12, 24 and 48 h after surgery Group A vs. Group B vs. Group C 1 h: 4.5 ± 0.8 vs. 4.4 ± 0.7 vs. 4.2 ± 0.9 (p = n.s.) 12 h: 6.1 ± 0.9 vs. 5.3 ± 1.0 vs. 5.2 ± 0.9 (*p* = 0.003, MCID-) 24 h: 7.0 ± 0.8 vs. 6.1 ± 0.7 vs. 6.4 ± 0.6 (*p* < 0.001, MCID -) 48 h: 4.6 ± 0.7 vs. 3.8 ± 0.5 vs. 3.6 ± 0.7 (*p* < 0.001, MCID -)	5

*ACB* dductor canal block, *APAP * acetaminophen, *b.i.d.* twice daily, *FNB* = femoral nerve block, *i.v.* intravenous,* i.m*. intramuscular, *MCID* = minimal clinically important difference, *mVAS * pain on visual analog scale during motion, *n.m.* not mentioned, *NRS *  numeric rating scale, *NSAIDs* nonsteroidal anti-inflammatory drugs, *p.o.* per os, *POD* postoperative day, *q.i.d.* four times daily, *RCT * randomized controlled trial, *rVAS* pain on visual analog scale at rest, *t.i.d.* three times daily, *TKA * total knee arthroplasty, *WOMAC* Western Ontario and McMaster Universities Osteoarthritis Index.

**Table 6:** Studies reporting clinical outcomes of nonsteroidal anti-inflammatory drugs

No.	Authors, Study type	Patients	Surgical procedure/ Anesthesia	Local infiltration	Basic analgesia	Intervention	Primary outcome and interpretation	Jadad score
1	Ma, 2021 (RCT) [74]	56	Bilateral TKA in general anesthesia	ACB	APAP 500 mg p.o. q.i.d., celecoxib 200 mg p.o. b.i.d. after surgery	Opioid-sparing group (parecoxib 40 mg i.v. b.i.d.) vs. Opioid-based group (morphine i.v.)	rVAS and mVAS at 6, 12, 24, 48 and 72 h (max. 10 mm, MCID = 2.26 mm reduction on VAS) mVAS Opioid-based vs. Opioid-sparing group at 24 h: 5.18 ± 2.47 vs. 3.68 ± 2.48 (*p* = 0.027, MCID -) at 72 h: 4.93 ± 2.23 vs. 3.50 ± 1.80 (*p* = 0.011, MCID -) at 12 h: 3.5 ± 0.8 vs. 3.1 ± 0.7 (*p* = 0.047, MCID -) No differences between rVAS at 24,48 and 72 h and no differences between mVAS at 48 h. Cumulative morphine consumption (mg) Opioid-based vs. Opioid-sparing group at 24 h: 20.99 ± 7.97 mg vs. 3.14 ± 2.62 mg (*p* < 0.001) at 48: 35.05 ± 12.46 mg vs. 4.21 ± 3.68 mg (*p* < 0.001) at 72 h: 45.69 ± 16.32 mg vs. 4.75 ± 4.33 mg (*p* < 0.001)	5
2	Laoruengthana, 2020 (RCT) [75]	100	Unilateral TKA in spinal anesthesia	Periarticular infiltration with bupivacaine and ketorolac vs. parecoxib	APAP 500 mg p.o. t.i.d.	ketorolac 30 mg i.v. b.i.d. until POD 2 vs. parecoxib 40 mg i.v. b.i.d. until POD 2	rVAS pain score at 6,12,18,24 and 48 h postoperatively (max. 10 cm, MCID = 1.4) rVAS ketorolac vs. parecoxib group: at 6 h: 2.38 ± 2.52 vs. 4.12 ± 2.86 (*p* < 0.001, MCID +) No differences between rVAS at 12,18,24 and 48 h. No differences in cumulative morphine consumption at 24 and 48 h.	5
3	Bian, 2018 (RCT) [76]	88	Unilateral TKA in general anesthesia	n.m.	n.m.	parecoxib 40 mg i.v. 30 min before surgery and then b.i.d. vs. Placebo	rVAS and mVAS in post-anesthesia care unit and at 6 h, 12 h, 24 h post-TKA and on POD 2,3,5,14 Significantly lower rVAS in the post-anesthesia care unit in the parecoxib group. No significant differences in the rVAS scores at other times between groups. No significant differences in the mVAS scores. (Continuous data not available)	5
4	Mammoto, 2020 (RCT) [77]	92	Unilateral TKA in general anesthesia	Femoral and sciatic nerve block with ropivacaine	n.m.	celecoxib group (400 mg p.o. 2 h post-TKA followed 6 h later by 200 mg; 200 mg b.i.d. until POD 7) vs. control group (celecoxib 400 mg p.o. orally at 9 AM on POD 2, followed 6 h later by 200 mg, then 200 mg b.i.d. until POD 7)	VAS pain score on POD 2 (max. 100 mm, MCID = 10 mm) VAS celecoxib vs. control group 18 mm (7–38) vs. 37 mm (20–52) (*p* = 0.0126, MCID +)	3
5	Xu, 2020 (RCT) [78]	160	Unilateral TKA in general anesthesia	n.m.	n.m.	CX group (celecoxib 200 mg p.o. b.i.d. from preoperative day 2 until POD 14) vs. TDB group (Transdermal buprenorphine 10 µg/h 2 days before operation until POD 19) + flurbiprofen 50 mg i.v. b.i.d.	rVAS at 2, 4, 6, 12, 24 and 48 h and on POD 3 and 7. mVAS at 12, 24 and 48 h and on POD 3, 7, 14 (MCID not defined) rVAS significantly lower at 2, 4, 6, 12, 24 and 48 h post-operatively in the TDB group compared with the CX group (MCID -) mVAS significantly lower at 12, 24 and 48 h post-operatively and on POD 3 in the TDB group (MCID -)	3
6	Zhuang, 2020 (RCT) [79]	246	Unilateral TKA in general anesthesia	n.m.	PCA with morphine i.v.	Study group (parecoxib 40 mg i.v. b.i.d. for the first 3 days post-TKA followed by celecoxib 200 mg p.o. b.i.d. for up to 6 weeks) vs. placebo	Cumulative opioid consumption at 2 weeks post-TKA parecoxib vs. placebo: 44 mg (36.3–82.5) vs. 101.8 mg (42.43-199.67) (*p* < 0.0001, MCID +)	5
7	Rawal, 2013 (RCT) [80]	776	Unilateral TKA in general or spinal anesthesia	No intraarticular analgesia	n.m.	Etoricoxib 120 mg p.o. q.d. vs. Etoricoxib 90 mg p.o. q.d. vs. Ibuprofen 1800 mg p.o. vs. Placebo	Mean Change from Baseline Average Pain Intensity at Rest over Days 1–3 (0–10 point-NRS) Placebo vs. Etoricoxib 90 mg: −3.39 (− 3.74, -3.04) vs. −3.93 (− 4.17, -3.69) (*p* = 0.018) Placebo vs. Etoricoxib 120 mg: −3.39 (− 3.74, -3.04) vs. −3.87 (− 4.11, -3.64) *p* = 0.009 Etoricoxib (both 120 mg and 90 mg) superior to placebo and non-inferior to ibuprofen.	5
8	Gong, 2013 (RCT) [81]	150	Unilateral TKA in general anesthesia	n.m.	PCA with morphine during hospital stay	Group A (celecoxib 300 mg p.o. b.i.d. and eperisone 50 mg p.o. t.i.d. for 14 days) vs. Group B (celecoxib 300 mg p.o. b.i.d and placebo p.o. t.i.d.) vs. Group C (placebo p.o. t.i.d.)	rVAS and mVAS on POD 1,3,7, 11,14 rVAS group A vs. B vs. C POD 7: 2.01 ± 1.61 vs. 2.72 ± 1.83 vs. 3.44 ± 1.58 (*p* = 0.0005, MCID-) POD 11: 1.16 ± 1.67 vs. 1.86 ± 1.58 vs. 2.59 ± 1.70 (*p* = 0.0004, MCID -) POD 14: 0.90 ± 1.73 vs. 1.64 ± 1.71 vs. 1.78 ± 1.62 (*p* = 0.03, MCID -) mVAS group A vs. B vs. C POD 3: 5.67 ± 1.23 vs. 6.32 ± 1.36 vs. 6.46 ± 1.43 (*p* = 0.0142, MCID -) POD 7: 5.0 ± 1.36 vs. 5.62 ± 1.41 vs. 5.74 ± 1.32 (*p* = 0.0254, MCID -) POD 11: 3.04 ± 1.27 vs. 3.56 ± 1.24 vs. 3.60 ± 1.32 (*p* = 0.0138, MCID -) Total dose of morphine until hospital discharge group A vs. B vs. C: 198.27 mg ± 55.36 vs. 225.62 mg ± 62.36 vs. 255.4 mg ± 59.67 (*p* = 0.0001 A vs. B)	5
9	Zhu, 2014 (RCT) [82]	100	Unilateral TKA in general anesthesia	Periarticular injection with morphine, adrenaline, ropivacaine	n.m.	parecoxib 40 mg i.v. at the completion of surgery and then b.i.d. until POD 3 vs. Placebo	Total dosage of morphine consumed in the first 24 h post-TKA (MCID = 20% reduction) Significantly less morphine consumption at 24 h in the parecoxib group (*p* < 0.05, MCID +)	5
10	Li, 2023 (RCT) [83]	106	Unilateral TKA in spinal anesthesia	n.m.	PCA with sufentanil and tramadol for 2 days	oxycodone-APAP 5/325 mg p.o. q.i.d. vs. celecoxib 200 mg p.o. b.i.d. until POD 3	rVAS and VAS with knee flexion on POD 0.5, 1,2,3,7 (max. 10 cm) rVAS in the oxycodone-APAP vs. celecoxib group POD 1: 3.7 ± 1.0 vs. 4.2 ± 1.0 (*p* = 0.015, MCID -) POD 2: 3.1 ± 0.9 vs. 3.5 ± 0.7 (*p* = 0.029, MCID -) POD 3: 3.0 ± 0.8 vs. 3.4 ± 0.8 (*p* = 0.015, MCID -) VAS with knee flexion in the oxycodone-APAP vs. celecoxib group POD 0.5: 5.2 ± 1.3 vs. 5.7 ± 1.3 (*p* = 0.038, MCID -) POD 1: 4.4 ± 1.2 vs. 5.1 ± 1.2 (*p* = 0.006, MCID -) POD 2: 3.9 ± 1.0 vs. 4.3 ± 1.1 (*p* = 0.015, MCID -) POD 3: 3.7 ± 0.8 vs. 4.1 ± 1.0 (*p* = 0.013, MCID -)	4
11	Berkowitz, 2021 (RCT) [84]	194	Unilateral TKA in spinal anesthesia	Local infiltration with bupivacaine and epinephrine	APAP 650 mg p.o., gabapentin 600 mg p.o. before surgery	morphine and then oxycodone + APAP 650 mg t.i.d. + meloxicam 30 mg i.v. or placebo i.v. prior to surgical incision, then q.d. while in hospital	total opioid use from end of surgery through 24 h (in i.v. morphine equivalents, MCID = 23% reduction) Meloxicam i.v. vs. Placebo group: 18.9 ± 1.32 vs. 27.7 ± 1.37 (*p* < 0.0001, reduction of 31.7%, MCID +)	5
12	Schroer, 2011 (RCT) [85]	107	Unilateral TKA in spinal anesthesia	n.m.	n.m.	celecoxib 400 mg p.o. before surgery and q.d. during hospitalization + celecoxib 200 mg p.o. b.i.d. for 6 weeks from discharge vs. placebo	Narcotic use by pill count in the first year postoperatively Celecoxib vs. Placebo group: 76.3 ± 55 (0-250) vs. 138 ± 117 (9-620) (*p* = 0.003)	5
13	Shao, 2020 (RCT) [86]	196	Unilateral TKA, anesthesia n.m.	n.m.	n.m.	Preoperative group (meloxicam 15 mg p.o. 24 h pre-TKA, 7.5 mg p.o. at 4 h, 24 h, 48 h, and 72 h post-TKA) vs. postoperative group (meloxicam 15 mg p.o. at 4 h post-TKA, then 7.5 mg p.o. at 24 h, 48 h, and 72 h post-TKA)	rVAS and VAS flexion at 24 h pre-TKA and at 6, 12, 24, 48, 72, and 96 h post-TKA. rVAS lower at 6, 12 and 24 h, while similar at 48, 72 and 96 h post-TKA in the preoperative vs. postoperative group. VAS at flexion lower at 6, 12, 24 h, while similar at 72 h and 96 h post-TKA in the preoperative vs. postoperative group (continuous data not available).	3
14	Tsuji, 2020 (RCT) [87]	79	Unilateral TKA in general anesthesia	Periarticular infiltration with levobupivacaine, methylprednisolone, morphine, epinephrine. No peripheral nerve block	n.m.	APAP 1800 mg during POD 1–14. FPP (flurbiprofen 40 mg) vs. SFPP (S-flurbiprofen 40 mg) every day until POD 14 vs. no patch	VAS on POD 1, 3, 5, 7, and 14 FPP vs. SFPP vs. Control group: POD 1: 24.4 ± 17.8 mm vs. 25.0 ± 18.8 mm vs. 39.4 ± 16.4 mm (*p* = 0.02 FPP vs. control and *p* = 0.035 SFPP vs. control) POD 3: 25.5 ± 21.0 mm vs. 23.3 ± 16.7 mm vs. 39.3 ± 15.4 (*p* = 0.042 FPP vs. control and *p* = 0.02 SFPP vs. control) No significant differences in VAS on POD 5, 7, and 14 between the groups.	3
15	Laoruengthana, 2022 (RCT) [88]	54	Bilateral TKA in spinal anesthesia	ketorolac group (bupivacaine + ketorolac) vs. bupivacaine	n.m.	ketorolac 30 mg i.v. t.i.d. for the first 48 h	VAS pain score at rest at 6, 12, 24, 48, 72, and 96 h after surgery and 2 and 6 weeks after surgery Ketorolac vs. Bupivacaine group 12 h: 4.25 ± 2.38 mm vs. 5.06 ± 2.48 mm (*p* = 0.04, MCID-) 24 h: 4.22 ± 1.94 mm vs. 5.3 ± 2.12 mm (*p* = 0.04, MCID-)	5
16	Xu, 2017 (intervention study) [89]	132	Unilateral TKA in general anesthesia	FNB and periarticular infiltration with methylprednisolone, epinephrine, ropivacaine	n.m.	PA group (celecoxib 200 mg p.o. b.i.d., tramadol 37.5 mg and APAP 325 mg p.o. t.i.d. 3 days post-TKA) vs. control. Postoperatively: tramadol/APAP 37.5 mg/325 mg p.o. t.i.d. 1 week, then stopped until POD10; parecoxib 40 mg i.v. b.i.d. until POD3, celecoxib 200 mg p.o. b.i.d. until POD4, then reduced to 200 mg q.d. for 10 days	rVAS and during maximal flexion and extension of the arthritic knee rVAS in the PA group vs. control: at 3 weeks: 3 cm (1–4) vs. 4 cm (2.25-5) (*p* = 0.013) at 6 weeks: 2 cm (1–3) vs. 2 cm (1–5) (*p* = 0.046) VAS during movement in the PA group vs. control: at 1 week: 3 cm (2–5) vs. 4 cm (3–6) (*p* = 0.015) at 3 weeks: 3 cm (2–5) vs. 4 cm (3-5.75) (*p* = 0.003) at 6 weeks: 2 cm (1–3) vs. 3 cm (2–5) (*p* = 0.003) at 3 months: 2 cm (1–2) vs. 2 cm (1–3) (*p* = 0.012)	MINORS 20/24

*ACB* adductor canal block, *APAP* acetaminophen, *b.i.d.* twice daily, *FNB* emoral nerve block, *i.v.* intravenous, *MCID* minimal clinically important difference, *mVAS* pain on visual analog scale during motion, *n.m.* not mentioned, *NRS* numeric rating scale, *PCA*  patient-controlled analgesia, *p.o.* per os, *POD * postoperative day, *q.d *  once daily, *q.i.d.* four times daily, *RCT*  randomized controlled trial, *rVAS*  pain on visual analog scale at rest, *TKA *  total knee arthroplasty.

### Duloxetine

Nine RCTs evaluating duloxetine as perioperative analgesia, out of which seven published in the last 5 years, with a total of 979 patients (Table 1) were selected. Only one study included cases of bilateral TKA in a single clinical setting [27] and only in three studies the surgical procedures were performed under general anesthesia [29, 32, 33]. The dose of prescribed duloxetine varied between 20 and 60 mg and the effects were compared to placebo in six RCTs [27–32]. The primary outcomes were defined as pain on a VAS or NRS scale at rest or movement [27, 29, 30, 32–34], as total morphine requirements [31] or both of them [28]. Regarding pain scores at rest and during movement, Rajani et al. [27], Yuan et al. [29] and Kim et al. [32] found a statistically significant difference in favor of the duloxetine group compared to placebo in the first postoperative week.

### Acetaminophen

Seven RCTs focusing on acetaminophen were included in the final analysis, focusing on 815 patients. (Table 2) Two RCTs failed to prove an adjunctive value of acetaminophen as part of multimodal analgesia by reporting similar pain scores and rescue analgesics consumption between acetaminophen and placebo [37, 38]. When added only to an NSAID, acetaminophen administered i.v. resulted in lower pain scores at 48 h in two RCTs [36, 42].

### Pregabalin, Gabapentin and nefopam

Zhou et al. [43] and Lee et al. [45] found a clinical improvement in the pain scores in the first 24 h postoperatively when pregabalin was added to celecoxib in the multimodal analgesia. In a RCT by Lunn et al. [49], gabapentin was not found to improve pain scores at 24 h postoperatively. A secondary follow-up of the same patients conducted by Petersen et al. [50] concluded that the perioperative use of gabapentin had also no influence on the occurrence of chronic postoperative pain after TKA. When used in combination with a femoral nerve block and an NSAID administered orally, the use of pregabalin did not show any significant improvement of pain scores in the acute postoperative phase [44, 47]. Nefopam, on the other side, showed a statistically significant improvement of pain scores and morphine consumption in the first 24 h postoperatively, however the results need to be critically evaluated regarding clinical significance [48, 51]. The detailed description of the abovementioned studies is presented in Table 3.

### Opioid analgesics

Out of the selected eight studies focusing on the perioperative use of opioids (Table 4), three involved the use of extended-release opioid formulations [52, 55, 59], two the use of topical or transdermal formulations [53, 54], one implied a combination of an opioid with acetaminophen [57], while other two assessed the efficacy of intraarticular opioid administration during TKA [56, 58]. Wang et al. [52] concluded that the preemptive use of oxycodone did not provide any clinical benefit with regards to postoperative consumption of rescue analgesia. However, fentanyl was non-inferior to morphine in an RCT by Hall et al. [54], therefore underlining the benefits of transdermal compared to oral opioid formulations.

### Corticosteroids

Studies focusing on the use of steroids as perioperative analgesia involved different administration routes, from oral administration [61, 66], to intravenous [62, 64, 65, 67, 68, 70, 72, 73] or a combination of intravenous and periarticular [60, 63, 69, 71] (Table 5). Shaw [61] and Cheng [66] concluded that the use of oral steroids resulted in lower VAS scores in the first four postoperative days and at 14 and 28 days after surgery, respectively. The combination of periarticular with intravenous steroids was proven to be more efficient compared to placebo in the RCT conducted by Wu [60], while the same combination provided lower pain scores also in comparison to the intravenous use of steroids, as reported in three RCTs [63, 67, 71]. The intravenous steroids were however superior to placebo [68, 70], but higher doses provided a supplementary benefit only in high-pain responders [65].

### Nonsteroidal anti-inflammatory drugs

Sixteen studies focused on the use of NSAIDs as part of multimodal analgesia, out of which fourteen involved the administration of cyclooxygenase-2 (COX 2)-inhibitors due to their better safety profile (Table 6). Five RCTs reported that parecoxib, celecoxib and etoricoxib are more efficient than placebo regarding postoperative pain scores and total morphine consumption within a time frame ranging from 24 h to 14 days post-TKA [79–82, 85]. On the other side, Bian et al. [76] concluded that the use of parecoxib in combination with morphine through patient-controlled analgesia resulted in a significant pain relief only in the first six postoperative hours. When compared to opioids, patients taking parecoxib reported lower pain scores in the first three days post-TKA, without reaching a clinically significant difference [74]. However, NSAIDs could be viewed as an adjuvant method in combination with opioids, as reported by Berkowitz [84], who concluded that the addition of meloxicam to morphine resulted in lower opioid consumption. Preoperative analgesia with NSAIDs proved also to be more efficient in pain treatment compared to the postoperative administration [77, 86].

**Fig. 4:**
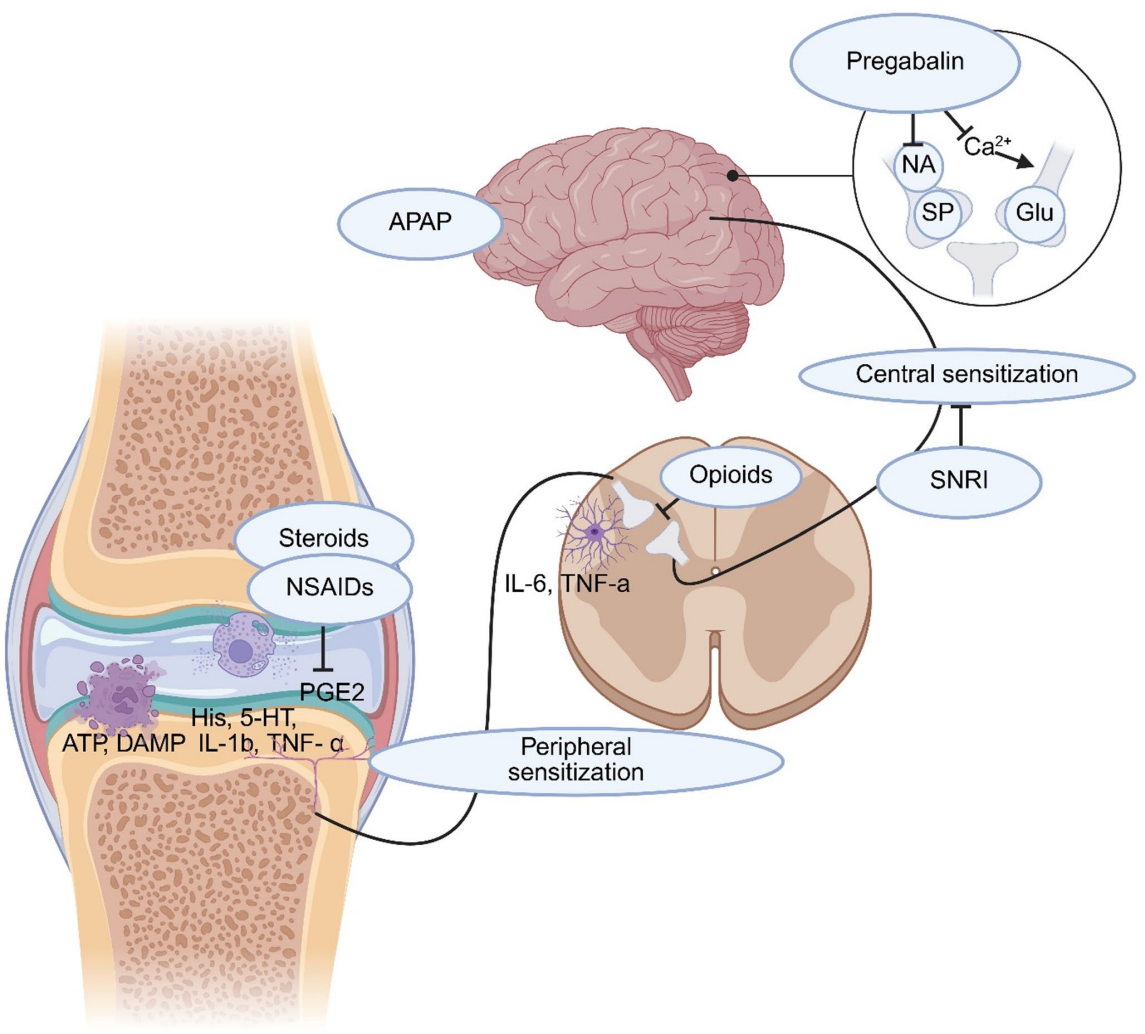
Mechanisms involved in central and peripheral sensitization. At the site of the injury, the mast cells are activated by the ATP and DAMP produced by necrotic cells, which favors the release of 5-HT and histamine. These substances directly activate the sensory neurons, leading to peripheral sensitization. As a consequence, the excitatory impulses transmitted to the posterior horn of the spinal cord leads to prolonged release of inflammatory markers and migration of astrocytes. (ATP = adenosine triphosphate, DAMP = danger-associated molecular patterns, His = histamine, 5-HT = 5-hydroxytryptamine, IL-1b = interleukin-1b, TNF-α = tumor necrosis factor alpha, PGE2 = prostaglandin E2, IL-6 = interleukin-6, NSAIDs = nonsteroidal anti-inflammatory drugs, SNRI = serotonin-norepinephrin reuptake inhibitor, NA = norepinephrine, SP = substance P, Glu = glutamate, APAP = acetaminophen. Created by the Authors in BioRender (https://BioRender.com/tbpru3w)

A summary of the identified mechanisms of action of the presented analgesics is illustrated in Fig. 4.

## Discussion

The main contribution of the present systematic review is the comparative illustration of multiple pharmacologic strategies used in the clinical setting with the aim of providing acute pain relief following TKA. One of the most important challenges encountered in the interpretation of the included studies resides in the evaluation of the clinical significance of the available published data, which differs from the simple achievement of the level of statistical significance.

Duloxetine improved pain scores at rest in the first 48 h postoperatively compared to placebo [27], as well as pain scores at rest and movement on the first postoperative day and after the first week respectively [32]. However, in a meta-analysis performed by Yang [90], duloxetine provided a slight pain reduction over a span of three days to eight weeks postoperatively, without showing an immediate analgesic effect. Moreover, duloxetine significantly reduced the cumulative opioid consumption in the first 48 h postoperatively [28, 31]. A meta-analysis conducted by Branton et al. [91] proved a significant reduction in the opioid consumption at 24 h, while Zorilla-Vaca et al. [92] recorded significant reductions in the opioid consumption at 24 and 48 h.

Acetaminophen is still a constant part of multimodal analgesic regimens, due to its low cost and attractive safety profile [93]. However, its efficiency in the reduction of postoperative pain is sustained only by moderate evidence [93]. Only two studies included in the present review demonstrated statistically significant better pain scores at 24 [41] and 48 h [36] postoperatively when comparing acetaminophen to placebo, however without reaching the threshold for clinical significance. This lack of benefit recorded by acetaminophen may also reside in the fact that all these studies included also NSAIDs in the pain management, which are known as potent and effective pain killers in osteoarthritis. Acetaminophen is used either as intravenous or as oral formulation, however in two meta-analyses conducted by Teng [94] and Sun [95], the intravenous administration was not superior in terms of pain management but implied significantly higher costs.

The role of pregabalin in the acute management of postoperative pain following TKA remains controversial. Only one study included in this review showed a significant difference in the postoperative pain scores when pregabalin was added to celecoxib [45]. A recent systematic review by Viderman et al. [96] concluded that pregabalin may aid in the reduction of postoperative pain, however the results may lack appropriate clinical significance. Buvanendran et al. [46] observed a clinically significant reduction in the incidence of chronic postoperative pain when pregabalin was added to the multimodal analgesic regimen, but a generalization of the benefit of using pregabalin is rather difficult to be made due to relatively scarce data available in the literature [96].

Opioids remain one of the most commonly prescribed analgesics following TKA, however the extent of their administration remains unknown due to the poor reporting of their use in clinical trials [97]. Moreover, the current literature emphasizes the need for promoting non-opioid regimens over opioids with the aim of decreasing addiction, overdose or adverse health effects [98]. Only one study included in this review [56] reported that the use of intraarticular morphine significantly decreases the cumulative postoperative opioid consumption, but without influencing pain scores at rest. This is in accordance with the evidence provided in a systematic review by Qi et al. [99], which acknowledges the benefit of the local infiltration with morphine in reducing opioid consumption, but also in improving pain scores at rest in the first 72 h.

The use of glucocorticoids following TKA is supported by their extensive anti-inflammatory properties, although with the simultaneous risk of high blood glucose, high blood pressure, wound infection, deep venous thrombosis [100] and even progression of the existing osteoarthritis [101]. A meta-analysis by Liang et al. [102] identified better pain scores when dexamethasone was added to multimodal analgesia, without notable differences between single and repeated doses of dexamethasone. However, the results were cautiously interpreted by the authors due to the unmet level of minimal important difference. Dexamethasone was not associated with increased risks of postoperative complications [102, 103], but its administration in diabetic patients is still controversial due to the exclusion of these category of subjects from RCTs [102]. Three studies included in our review found clinically important differences in pain scores at 24 h when intravenous steroids were administered [60, 68, 70], while one study [64] reported better pain outcomes after 14 days of oral prednisone administration.

NSAIDs are highly effective agents in treating postoperative pain, with an acceptable safety profile, when taking associated comorbidities of the patients in consideration. In our review, parecoxib and meloxicam showed a clinically relevant opioid-sparing effect [79, 82, 84, 85]. Moreover, parecoxib and celecoxib provided better pain scores in the immediate postoperative phase compared to placebo [76, 77]. Fillingham et al. [104] reported in a meta-analysis, that the use of COX2-inhibitors or non-selective NSAIDs is a highly efficient treatment strategy for pain management following TKA. Hong et al. [105] reported that the use of parecoxib had the highest efficiency in the first 24 postoperative hours, whilst not differing from placebo at 48 h.

Some important limitations of the available RCTs and meta-analyses is the inclusion of both total knee and total hip arthroplasties, as well as the use of variable types of anesthesia. Since TKA is regarded as a more painful procedure compared to total hip arthroplasty, the results of different analgesic regimens applied in both types of surgery are difficult to be generalized for TKA. Moreover, it is difficult to make a conclusive statement regarding the effect of general or spinal anesthesia on the degree of postoperative pain following TKA. Current guidelines favor the use of spinal over general anesthesia in TKA, based on the reduced postoperative complication rates, however only with a low level of evidence [106].

This systematic review offers the clinician a structured and broad synthesis of the available therapeutic strategies for reducing pain following TKA. Based on our findings, NSAIDs remain one of the first choices in the perioperative pain management following TKA, with COX2-inhibitors such parecoxib exhibiting satisfactory pain control. Due to the limitative use of NSAIDs based on dose-dependent adverse effects, acetaminophen may be used as an adjuvant agent. Opioids may still be prescribed as rescue analgesics for a clearly definite timeframe, while the use of other substances such as duloxetine warrants further investigation.

### Limitations

The present review has some limitations. First, the included studies were mostly heterogenous in design, which makes the interpretation of results rather difficult. Moreover, some outcomes were assessed only in a few studies, therefore conclusions cannot be drawn. Lastly, in order to strengthen the validity of results, we included only RCTs which addressed the review question as primary outcome; however, this may exclude other available studies.

## Conclusion

Based on the currently available data, the appropriate pain management following TKA emphasizes the promotion of non-opioid treatment strategies. The main treatment option remains the use of NSAIDs, followed by acetaminophen, based on its low cost and acceptable safety profile. Duloxetine seems a new emerging possibility, but still lacks validation by large RCTs. Opioids are still highly prescribed, but fail to prove an evident clinical benefit, while steroids and gabapentinoids should not be a standard treatment strategy due to adverse effects and modest clinical impact. Further larger RCTs are required to better define a more appropriate and complex multimodal analgesic protocol following TKA.

## Supplementary Information

Below is the link to the electronic supplementary material.

Supplementary Material 1

## Data Availability

No datasets were generated or analysed during the current study.
