# Supplementary material for: Pharmacologic pain management strategies for reducing postoperative pain in total knee arthroplasty: a systematic review from molecular mechanisms to clinical efficiency
Source: Arch Orthop Trauma Surg. 2025 Sep 1;145(1):432. doi: 10.1007/s00402-025-06049-7 (PMC12401767; doi:10.1007/s00402-025-06049-7)
Supplement: Supplementary file 1 — Supplementary Material 1 [file 402_2025_6049_MOESM1_ESM.docx]

**Additional Information 1. Search strategy**

**Pubmed: 486 results**

(("duloxetine"[Title] OR "acetaminophen"[Title] OR "paracetamol"[Title] OR "gabapentin"[Title] OR ("anti inflammatory agents non steroidal"[Pharmacological Action] OR "anti inflammatory agents, non steroidal"[MeSH Terms] OR ("anti inflammatory"[All Fields] AND "agents"[All Fields] AND "non steroidal"[All Fields]) OR "non-steroidal anti-inflammatory agents"[All Fields] OR "nsaid"[All Fields] OR "nsaids"[All Fields] OR "nsaid s"[All Fields]) OR ("analgesics opioid"[Pharmacological Action] OR "analgesics, opioid"[MeSH Terms] OR ("analgesics"[All Fields] AND "opioid"[All Fields]) OR "opioid analgesics"[All Fields] OR "opioid"[All Fields] OR "opioids"[All Fields] OR "opioid s"[All Fields]) OR ("steroidal"[All Fields] OR "steroidals"[All Fields] OR "steroidic"[All Fields] OR "steroids"[MeSH Terms] OR "steroids"[All Fields] OR "steroid"[All Fields]) OR "pregabalin"[Title]) AND "total knee arthroplasty"[Title]) AND ((clinicalstudy[Filter] OR clinicaltrial[Filter] OR clinicaltrialphasei[Filter] OR clinicaltrialphaseii[Filter] OR multicenterstudy[Filter] OR observationalstudy[Filter] OR randomizedcontrolledtrial[Filter]) AND (2010/1/1:2024/9/1[pdat]) AND (english[Filter]))

**Scopus: 378 results**

( TITLE ( duloxetine ) OR TITLE ( acetaminophen ) OR TITLE ( paracetamol ) OR TITLE ( pregabalin ) OR TITLE ( gabapentin ) OR TITLE-ABS-KEY ( opioid ) OR TITLE-ABS-KEY ( steroid ) OR TITLE-ABS-KEY ( nsaid ) AND TITLE ( total AND knee AND arthroplasty ) AND NOT TITLE-ABS-KEY ( total AND hip AND arthroplasty ) ) AND PUBYEAR > 2009 AND PUBYEAR < 2025 AND ( LIMIT-TO ( DOCTYPE , "ar" ) ) AND ( LIMIT-TO ( SUBJAREA , "MEDI" ) OR LIMIT-TO ( SUBJAREA , "BIOC" ) OR LIMIT-TO ( SUBJAREA , "HEAL" ) OR LIMIT-TO ( SUBJAREA , "NURS" ) OR LIMIT-TO ( SUBJAREA , "PHAR" ) OR LIMIT-TO ( SUBJAREA , "NEUR" ) OR LIMIT-TO ( SUBJAREA , "MULT" ) OR LIMIT-TO ( SUBJAREA , "IMMU" ) ) AND ( LIMIT-TO ( LANGUAGE , "English" ) ) AND ( LIMIT-TO ( EXACTKEYWORD , "Total Knee Arthroplasty" ) OR LIMIT-TO ( EXACTKEYWORD , "Arthroplasty, Replacement, Knee" ) OR LIMIT-TO ( EXACTKEYWORD , "Postoperative Pain" ) OR LIMIT-TO ( EXACTKEYWORD , "Knee Replacement" ) OR LIMIT-TO ( EXACTKEYWORD , "Pain, Postoperative" ) OR LIMIT-TO ( EXACTKEYWORD , "Analgesics, Opioid" ) OR LIMIT-TO ( EXACTKEYWORD , "Narcotic Analgesic Agent" ) OR LIMIT-TO ( EXACTKEYWORD , "Analgesia" ) OR LIMIT-TO ( EXACTKEYWORD , "Morphine" ) OR LIMIT-TO ( EXACTKEYWORD , "Opiate" ) OR LIMIT-TO ( EXACTKEYWORD , "Paracetamol" ) OR LIMIT-TO ( EXACTKEYWORD , "Visual Analog Scale" ) OR LIMIT-TO ( EXACTKEYWORD , "Pain Management" ) OR LIMIT-TO ( EXACTKEYWORD , "Postoperative Analgesia" ) OR LIMIT-TO ( EXACTKEYWORD , "Pain Measurement" ) OR LIMIT-TO ( EXACTKEYWORD , "Pain Assessment" ) OR LIMIT-TO ( EXACTKEYWORD , "Knee" ) OR LIMIT-TO ( EXACTKEYWORD , "Oxycodone" ) OR LIMIT-TO ( EXACTKEYWORD , "Pain" ) OR LIMIT-TO ( EXACTKEYWORD , "Knee Osteoarthritis" ) OR LIMIT-TO ( EXACTKEYWORD , "Celecoxib" ) OR LIMIT-TO ( EXACTKEYWORD , "Total Knee Replacement" ) OR LIMIT-TO ( EXACTKEYWORD , "Patient Controlled Analgesia" ) OR LIMIT-TO ( EXACTKEYWORD , "Ketorolac" ) OR LIMIT-TO ( EXACTKEYWORD , "Tramadol" ) OR LIMIT-TO ( EXACTKEYWORD , "Drug Efficacy" ) OR LIMIT-TO ( EXACTKEYWORD , "Analgesic Agent" ) OR LIMIT-TO ( EXACTKEYWORD , "Knee Arthroplasty" ) OR LIMIT-TO ( EXACTKEYWORD , "Dexamethasone" ) OR LIMIT-TO ( EXACTKEYWORD , "Nonsteroid Antiinflammatory Agent" ) OR LIMIT-TO ( EXACTKEYWORD , "Arthroplasty" ) OR LIMIT-TO ( EXACTKEYWORD , "Analgesics" ) OR LIMIT-TO ( EXACTKEYWORD , "Pregabalin" ) OR LIMIT-TO ( EXACTKEYWORD , "Numeric Rating Scale" ) OR LIMIT-TO ( EXACTKEYWORD , "Gabapentin" ) OR LIMIT-TO ( EXACTKEYWORD , "Opioids" ) OR LIMIT-TO ( EXACTKEYWORD , "Steroid" ) OR LIMIT-TO ( EXACTKEYWORD , "Hydromorphone" ) OR LIMIT-TO ( EXACTKEYWORD , "Opioid" ) OR LIMIT-TO ( EXACTKEYWORD , "Analgesia, Patient-Controlled" ) OR EXCLUDE ( EXACTKEYWORD , "Regional Anesthesia" ) OR EXCLUDE ( EXACTKEYWORD , "Adductor Canal Block" ) OR EXCLUDE ( EXACTKEYWORD , "Nausea" ) OR EXCLUDE ( EXACTKEYWORD , "Vomiting" ) OR EXCLUDE ( EXACTKEYWORD , "Pruritus" ) OR EXCLUDE ( EXACTKEYWORD , "Dizziness" ) OR EXCLUDE ( EXACTKEYWORD , "Propofol" ) OR EXCLUDE ( EXACTKEYWORD , "General Anesthesia" ) OR EXCLUDE ( EXACTKEYWORD , "Meta Analysis" ) OR EXCLUDE ( EXACTKEYWORD , "Femoral Nerve Block" ) OR EXCLUDE ( EXACTKEYWORD , "Local Infiltration Analgesia" ) OR EXCLUDE ( EXACTKEYWORD , "Deep Vein Thrombosis" ) OR EXCLUDE ( EXACTKEYWORD , "Ondansetron" ) OR EXCLUDE ( EXACTKEYWORD , "Anesthesia" ) OR EXCLUDE ( EXACTKEYWORD , "Tranexamic Acid" ) OR EXCLUDE ( EXACTKEYWORD , "Nausea And Vomiting" ) OR EXCLUDE ( EXACTKEYWORD , "Anesthesia, Spinal" ) ) AND ( LIMIT-TO ( SRCTYPE , "j" ) )

**Embase, MEDLINE, Preprints: 198 results**

('duloxetine'/exp OR 'duloxetine' OR 'paracetamol':ti OR 'pregabalin':ti OR 'gabapentin':ti OR 'nonsteroid antiinflammatory agent':ti,ab,kw OR 'opiate':ti,ab,kw OR 'steroid':ti,ab,kw) AND 'total knee arthroplasty':ti
